# Households catastrophic health expenditure among older people with noncommunicable diseases: A cross‐sectional study from Iran

**DOI:** 10.1002/hsr2.1992

**Published:** 2024-03-18

**Authors:** Sayed Amir Masoud Meshkat, Sayed Saeed Kassaeian, Navid Danaei, Farid Gharibi

**Affiliations:** ^1^ School of Medicine Semnan University of Medical Sciences Semnan Iran; ^2^ Department of Community Medicine, School of Medicine Semnan University of Medical Sciences Semnan Iran; ^3^ Department of Pediatrics, School of Medicine Semnan University of Medical Sciences Semnan Iran; ^4^ Social Determinants of Health Research Center Semnan University of Medical Sciences Semnan Iran

**Keywords:** catastrophic health costs, elderly, financial protection, noncommunicable diseases

## Abstract

**Background and Aims:**

Noncommunicable diseases are one of the main difficulties faced by older adults in many countries. The study aimed to investigate the households' catastrophic health expenditure (CHE) among older people with noncommunicable diseases.

**Methods:**

This cross‐sectional study was conducted between October and December 2022 in Semnan, Iran, with the participation of 400 older individuals suffering from diabetes and hypertension. The Content Validity Ratio (which is calculated based on necessity criterion) and Content Validity Index (which calculated based on relevance, transparency, and simplicity criteria) values of 0.94 and 0.89, respectively, confirmed the content validity of the researcher‐made questionnaire. The occurrence of CHE was estimated using the World Health Organization's rule of “allocating at least 40% of non‐food household costs to healthcare.” Due to the qualitative nature of the data, the *χ* ^2^ test was utilized to assess the statistical association between demographic and background variables and occurrence of CHE.

**Results:**

Older people with diabetes and hypertension had an annual direct medical costs of $821 averagely, which includes 9.7% for diagnosis, 23.9% for doctor visits, and 66.4% for treatment. Direct medical costs account for around 0.26 of nonfood costs, with 12.5% of seniors facing high medical charges. Age, marital status, type of basic health insurance, diabetes, the time elapsed since initial diabetes diagnosis, the severity of diabetes complications, and the development of diabetes‐related visual impairments, are associated with CHE (*p* < 0.05).

**Conclusion:**

While the prevalence of CHE among elderly individuals with diabetes and hypertension is reasonably manageable, targeted promotional efforts are still necessary to protect those at high risk.

## 1 INTRODUCTION

More individuals are reaching old age due to the rise in life expectancy in the contemporary era, which is a result of societies' economic, social, and health‐related advancements. Extensive biological changes at this age are often associated with an increased risk of noncommunicable diseases and decrease in functional capacities.^1^ In contrast, population aging refers to the increase in the average age of a society's citizens due to a rise in life expectancy and a decline in the birth rate. This phenomenon has widespread negative social, economic, and health consequences for societies, including a rise in overhead costs, a reduction in production capacity, and an increase in healthcare costs for healthcare systems.^2^

According to United Nations population estimates, the world's aged population will expand from approximately 900 million in 2015 to 1.3 billion in 2030 (16% of the world's population) and 2 billion in 2050 (22% of the world's population).^3^ This global increasing trend in Iran is also clearly visible in such a way that increasing from 7.2% to 8.2% between 2006 and 2011, Iran's old population is projected to reach 10.5% and 21.7% in 2025 and 2050, respectively.^4^ Other projections indicate that by 2050, 33% of Iran's population will be aged, compared to 21% in other nations.^5^ Iran lacks the essential preparation to deal with this important transition in all societal dimensions, particularly the economic and health aspects.^4^

In Iran, 72.8% of the elderly suffer from arthritis, 55.2% from excess body fat, 53% from hypertension, 49.8% from impaired visual, 43.9% from a history of infectious diseases, 40.1% from sleep disorders, 36.1% from heart disease, 30.6% from urinary problems, 24.6% from mental‐psychological issues, 22.5% from respiratory issues, 14.8% from type 2 diabetes, 18% from a history of falls, and 12.0% from having a history of stroke.^6^ Since more than 72% of the old population is either illiterate or has a low level of education, this may provide a significant challenge when it comes to educating the elderly about self‐care.^4^

One of the most imperative shortcomings in Iran in facing this notable demographic transition is financial issues because 63% of the elderly lack the finances to meet their economic needs especially their healthcare costs, and 17% are unemployed and without income.^4^ To effectively manage the health negative effects of aging population and its notable costs, it is crucial to have an efficient and adaptable healthcare system.^7^ One of the most important indicators in a society is the catastrophic health expenditure (CHE) which represents the level of social support and justice for citizens.^8^

Currently, 4.7% of Iranian households are experiencing CHE and this number is steadily increasing^8^; due to problems such as insufficient per capita health budget, inefficient structure of health insurance, high and unfair amount of out‐of‐pocket payments, inability to create universal health coverage, centralized management style, and prioritizing treatment over prevention.^8^, ^9^ This issue is prevalent in other developing nations, such as India, where CHE surged from 12.4% in 1993 to 24.9% in 2014.^10^ Also, Iranian studies^8^, ^10^ cite the presence of an elderly household member as one of the reasons for their CHE; this situation is also evident in other nations^11^ because the health costs imposed on the elderly are at least 50% higher than those imposed on younger individuals.^12^ Increased rates and severity of noncommunicable diseases like heart disease, renal disease, diabetes, and cancer significantly contribute to the higher healthcare costs incurred by the elderly population.^13^ Given lack of similar studies, this study aimed to assess the households' CHEs among older people with noncommunicable diseases.

## 2 METHODS

### 2.1 Study type and participants

This cross‐sectional study involving 400 older individuals with diabetes and hypertension (the most prevalent noncommunicable diseases) was accomplished in Semnan city in October 2022. The inclusion criteria for the study participants were being at least 60 years old, suffering from diabetes or hypertension, and having received treatment for these conditions at least a year before participation in the present study.

### 2.2 Sample size and sampling method

The data on the cost of recent years were collected and examined to investigate the magnitude and determinants of CHE. The study sample size was calculated using the following formula:

$$n={\left(\frac{\mathrm{zs}}{d}\right)}^{2}={\left(\frac{1.96*0.5}{0.05}\right)}^{2}=\left(\frac{0.9604}{0.0025}\right)=384.16.$$

The *Z*‐score value at a given degree of confidence is represented by *z*, projected changes in responses by *s*, and the margin of error by *d*. The sample size for the study was determined at a confidence level of 95%, with a prediction change of 0.5 and an error margin of 0.05, and based on the recommendation of some scientific sources to increase the sample size by 5%, 400 elderly individuals were randomly selected, and their information was entered into the questionnaire.

### 2.3 Instruments

A questionnaire created by the researchers was used in the investigation. The questionnaire was created using information acquired from interviews with geriatrics, health management, and geriatric medicine professionals, as well as data from comparable studies. Diabetes and hypertension costs incurred by the elderly were classified as diagnostic, visit, and treatment costs. The demographic and background variables that may influence the CHE were then determined based on expert comments and comparative studies, and a prototype questionnaire was created by integrating them. The validity of the questionnaire was then assessed in terms of content and form using the opinions of 10 experts. All questionnaire items were evaluated using the four spectrum criteria of necessity, relevance, transparency, and simplicity, and the Content Validity Ratio (CVR) and Content Validity Index (CVI) indices were calculated based on the outcome. Experts' feedback on the survey questions' design was also used to confirm the instrument's face validity.^14^, ^15^ The CVR index was developed based on the responses to the necessity index questions. In addition, the CVI index was calculated using the answers to three more criteria using the following formula^14^, ^15^:

$$\text{CVR}\;=\frac{nE-\frac{N}{2}}{\frac{N}{2}}.$$

*n*E is the number of experts who chose the spectrum's positive alternatives, and *N* is the total number of experts. The acceptability score in this study was established at 0.62 due to the participation of 10 experts in preparing the questionnaire.^14^, ^15^ The final questionnaire contained 87 items, 32 dedicated to independent demographic/background characteristics, 45 to cost aspects, 4 to the income of the elderly and their families, and 6 to the elderly's perspective of cost pressure and its implications. The final instrument's validity was proved by expert scores of 0.94 and 0.89 for CVR and CVI indicators, respectively.

### 2.4 Data collection and data analysis

The direct medical costs of the elderly were gathered through interview with the elderly. In this way, all the questions defined in the questionnaire regarding the types of diagnostic, treatment and rehabilitation costs were asked orally from the participants and their desired answers were entered in the questionnaire. Also, to ensure the accuracy of the cost information given by the participants, the services received by them in government centers and the paid costs recorded in the care systems were also carefully examined.

Next, the proportion of a household's nonfood costs allocated to direct medical costs was assessed, and costs over 40% were deemed catastrophic.^16^ In addition to studying basic demographics (gender, age, work status), we looked at background factors (primary and supplemental health insurance, location of care) and variables concerning condition duration, treatment status, and complications. The results for quantitative and qualitative variables were presented as the mean (standard deviation) and the frequency (percentage), respectively. Due to the qualitative nature of the data, the *χ* ^2^ test (as two‐sides hypothesis) was utilized to evaluate the statistical association between demographic and background variables and the occurrence of CHE. All of occurred analysis were done with version 22 of SPSS software.

### 2.5 Ethical considerations

All seniors who volunteered to participate in the study submitted their consent, and their participation was entirely voluntary. The Semnan University of Medical Sciences Ethics Committee granted ethical approval (IR.SEMUMS.REC.1400.211), assuring that the senior participants' right to privacy and dignity were respected throughout the research process. Although, the informed consents was obtained from participants of study.

## 3 RESULTS

The participants' average age was 67.63 (±4.09), ranging from 61 to 84. Ninety percent were between the ages of 60 and 74 and were labeled as young old; 10% were between 75 and 90 and were labeled as elderly or middle old, and none were older than 90 (oldest old). The participants were divided equally between the sexes, most married. Most elderly have less than a high school education and are predominantly retirees or homemakers. All participants were urban residents and natives of the center of the province. They all had basic health insurance; most were covered by social security and medical services. Also, most participants had supplemental health insurance. Diabetes and hypertension affected 60% and 80% of the population, respectively, with around 40% suffering from both illnesses. In addition, most older individuals received essential health care and treatment from the public and private facilities (Table 1).

**Table 1:** Demographic and background characteristics of the elderly.

Demographic variables	Category	Frequency	%
Age	60–74 years old (young old)	362	90.5
	75–90 years old (middle old)	38	9.5
	More than 90 years old (oldest old)	0	0
Gender	Male	198	49.5
	Female	202	50.5
Marital status	Married	343	85.75
	Deceased spouse	57	14.25
Educational status	Illiterate	46	11.5
	Below high school diploma	157	39.25
	High school diploma	126	31.5
	Associate's and bachelor's degrees	59	14.75
	Master's degree	12	3
Occupational status	Office employee	8	2
	Worker	2	0.5
	Self‐employed	11	2.75
	Retired	228	57
	Housekeeper	151	37.75
Nativity	Center of province	400	100
	Other places of province	0	0
Living place	Urban dwellers	400	100
	Rural dwellers	0	0
Benefiting basic insurance	Yes	400	100
	No	0	0
Type of basic insurance	Social security	259	64.75
	Treatment services	108	27
	Armed forced	23	5.75
	Banks	4	1
	Petroleum	6	1.5
Benefiting supplementary insurance	Yes	362	90.5
	No	38	9.5
Type of noncommunicable illness in the elderly	Diabetes	238	59.5
	Hypertension	316	79
	Diabetes and hypertension	152	38
Place of receiving care	Governmental center	55	14.25
	Private center	7	1.25
	Integration of private and public centers	338	84.5

One‐third of the elderly with diabetes were diagnosed between 5 and 10 years, and the remaining third were diagnosed between 10 and 20 years after the commencement of the ailment. Most patients began receiving proper care following the first disease diagnosis. Two‐thirds of them are also continually under the care of a physician and receive services from general practitioners, internal specialists, and endocrinologists in similar proportions. Diabetes affects more than a quarter of the elderly, with eye, kidney, and feet vascular problems as the most common (Table 2).

**Table 2:** Background variables related to diabetes.

Demographic variables	Category	Frequency	%
The time elapsed since the initial diagnosis of the disease	Less than 5 years	32	13.5
	5–9 years	78	32.8
	10–19 years	80	33.6
	20 years and more	48	20.2
The time interval between the initial diagnosis and the start of care	Immediately	223	93.7
	Less than 1 year	8	3.4
	1–4 years	5	2.1
	5–10 years	2	0.8
Controlling of the disease	Continuous	163	68.5
	Periodic	60	25.2
	In the event of an sever problem	15	6.3
The doctor in charge of the treatment	General practitioner	90	37.8
	Internal specialist	77	32.3
	Endocrinologist	63	26.5
	Urologist and nephrologist	8	3.4
Occurring serious complications	Yes	62	26.1
	No	176	73.9
Type of complications	Nephropathy	26	10.9
	Retinopathy	33	13.9
	Diabetic foot	18	7.6
	Amputation	2	0.8

More than a third of the elderly with hypertension have had the disease for 5–9 years, and more than a quarter have had it for 10–20 years. Most seniors began receiving care following the first diagnosis of their condition, but less than half of them are consistently under a physician's care. Approximately two‐thirds of the elderly are under the care of a general practitioner, while the remainder is under the care of a cardiologist and an internal medicine specialist. About one‐fifth of the elderly suffer from significant hypertension consequences, with heart complications accounting for the highest proportion (Table 3).

**Table 3:** Background variables related to hypertension.

Demographic variables	Category	Frequency	%
The time elapsed since the initial diagnosis of the disease	Less than 5 years	67	21.2
	5–9 years	118	37.3
	10–19 years	85	26.9
	20 years and more	46	14.6
The time interval between the initial diagnosis and the start of care	Immediately	296	93.7
	Less than 1 year	14	4.4
	1–4 years	4	1.3
	5–10 years	2	0.6
Controlling of the disease	Continuous	153	48.4
	Periodic	109	34.5
	In the event of an sever problem	52	16.5
	Not under care	2	0.6
The doctor in charge of the treatment	General practitioner	198	63.1
	Cardiologist	66	21
	Internal specialist	50	15.9
Occurring serious complications	Yes	60	19
	No	256	81
Type of complications	Cardiac compactions	45	14.2
	Myocardial infarction	12	3.8
	Stroke	4	1.3

According to a financial burden estimate, persons with diabetes and hypertension have direct medical costs totaling $821. This sum is divided as follows: $80 (9.7%) is spent on diagnostic services, $196 (23.9%) is spent on office visits, and $545 (66.4%) is spent on medicine. Most diagnostic costs are allocated to laboratory and ultrasound services; most visit costs are allocated to general practitioners and traditional healers, and most treatment costs are allocated to medication (Table 4).

**Table 4:** Amount and type of direct medical expenses imposed.

Domain	Type of costs	Minimum		Maximum		Average (standard deviation)	
		IRR	USD	IRR	USD	IRR	USD
Diagnosing services	Laboratory services	0	0	21,000,000	500	1,827,500 (±2,625,760)	43.51 (±62.52)
	Sonography	0	0	4,200,000	100	827,500 (±1,146,100)	19.71 (±27.29)
	CT scan	0	0	3,500,000	83.33	35,000 (±349,110)	0.83 (±8.31)
	MRI	0	0	15,000,000	357.14	400,000 (±1,993,700)	9.52 (±47.47)
	Other diagnosing costs	0	0	10,000,000	238.09	250,000 (±1,287,740)	5.95 (±30.66)
	*Sum of diagnosing costs*	*0*	*0*	*24,500,000*	*583.33*	*3,340,000 (*±4,353,540*)*	*79.52 (*±103.65*)*
Visiting	General physician	0	0	75,000,000	1785.71	3,885,250 (±53,027,650)	92.51 (±1262.56)
	Internist and endocrinologist	0	0	4,500,000	107.14	937,250 (±1,203,580)	22.32 (±28.66)
	Ophthalmologist	0	0	3,000,000	71.43	438,000 (±674,090)	10.43 (±16.05)
	Urologist and nephrologist	0	0	4,800,000	114.28	251,250 (±732,190)	5.98 (±17.43)
	Orthopedic and general surgeon	0	0	2,000,000	47.62	42,000 (±236,880)	1 (±5.64)
	Psychiatrist and psychologist	0	0	2,500,000	59.52	26,500 (±232,410)	0.63 (±5.53)
	Cardiologist	0	0	120,000,000	2857.14	1,130,000 (±849,407)	26.91 (±)
	Neurologist	0	0	3,000,000	71.43	32,000 (±272,290)	0.76 (±6.48)
	Nutritionists	0	0	850,000	20.23	46,750 (±154,580)	1.11 (±3.68)
	Traditional medicine	0	0	10,000,000	238.09	1,402,500 (±2,231,460)	33.39 (±53.13)
	Other care providers	0	0	3,500,000	83.33	45,000 (±368,990)	1.07 (±8.78)
	*Sum of visiting costs*	*0*	*0*	*75,000,000*	*1787.71*	*8,236,500 (±53,513,190)*	*196.11 (±1274.12)*
Treatment services	Hospitalization services	0	0	60,000,000	1428.57	646,000 (±4,933,460)	15.38 (±117.46)
	Medication	0	0	150,000,000	3571.43	15,868,500 (±13,801,450)	377.82 (±328.60)
	Dialysis	0	0	360,000,000	8571.42	1,800,000 (±25,455,840)	42.85 (±606.09)
	Informal/Under table payments	0	0	50,000,000	1190.47	250,000 (±3,535,530)	5.95 (±84.18)
	Physiotherapy	0	0	25,000,000	595.28	699,490 (±2,465,710)	16.50 (±58.70)
	Purchasing of prosthesis	0	0	500,000,000	11904.76	3,500,000 (±38,012,820)	83.33 (±905.07)
	Other treatments costs	0	0	30,000,000	714.28	150,000 (±2,121,320)	3.57 (±50.51)
	*Sum of treatment costs*	*0*	*0*	*574,000,000*	13,666.66	*22,907,000 (*±*51,547,690)*	*545.40 (*±*1227.32)*
Sum of costs		0	0	753,600,000	17,942.85	34,483,500 (±74,059,330)	821.03 (±173.32)

The cost of diagnosing diabetes is somewhat greater than that of diagnosing hypertension in senior patients with noncommunicable illnesses, but the costs would be much higher if both conditions are diagnosed concurrently. Examining the cost of visits for two illnesses reveals that the cost of visits for hypertension is more than 2.2 times that of diabetes, even though, in the case of comorbidity, this cost is only marginally more than that of diabetes. In addition, the cost of treating diabetes is more than that of treating hypertension, and it will rise again (albeit not much) in the event of comorbidity. In general, the direct medical costs in comorbidity of diabetes and hypertension will be more than the cost of one of the diseases. Also, the costs incurred by the elderly due to diabetes are greater than those incurred due to hypertension (Table 5).

**Table 5:** Comparison of different direct medical costs imposed on the elderly with noncommunicable diseases.

Costs	Diabetes		Hypertension		Comorbidity (diabetes & hypertension)	
	IRR	USD	IRR	USD	IRR	USD
Diagnosing costs	3,778,990 (±4,744,210)	89.97 (±112.95)	3,581,640 (±4,646,240)	85.27 (±110.62)	4,550,000 (±5,406,100)	108.33 (±128.71)
Visiting costs	4,271,420 (±2,843,000)	101.70 (±67.69)	9,453,480 (±60,172,480)	225.08 (±1432.67)	4,613,810 (±2,895,110)	109.85 (±68.93)
Treatment costs	26,440,330 (±41197260)	629.53 (±980.88)	23,283,540 (±55,502,720)	554.37 (±1321.49)	29,392,100 (±45,208,780)	699.81 (±1076.39)
Direct medical costs	34,490,750 (±43,743,820)	821.20 (±1041.51)	36,318,670 (±81,421,950)	864.73 (±1938.61)	38,555,920 (±48,177,850)	917.99 (±1147.09)

The mean annually income of the elderly and their families is estimated to be $20,218; $4101 is allocated to nonfood costs. The proportion of nonfood costs allocated to direct medical costs is 0.26%, while 12.5% of the elderly experience CHE. In addition, CHE spending rates are 16.8%, 10.1%, and 14.5%, respectively, among the elderly with diabetes, hypertension, or both.

CHE are associated with several demographic and background factors, including age, marital status, type of basic health insurance, diabetes, time passed after the first diagnosis, occurrence of diabetes complications, and development of eyesight difficulties owing to diabetes (*p* > 0.05). As a result, the prevalence of CHE in young old, elderly individuals is low; married elderlies spend less than widower elderly, and the elderly covered by armed forces, banks, and oil companies incur fewer costs than the elderly covered by other types of insurance. Furthermore, the elderly with diabetes incur more CHE than the elderly without diabetes; the elderly with more than 10 years of diabetes history face higher costs than the elderly with less diabetes history; and the elderly with diabetes complications, particularly visual complication, face higher costs than the elderly without these complications (Table 6).

**Table 6:** Statistical relationship between demographic/background variables with the occurrence of CHE.

Variable	Category	Incidence (%)	Significance (*p*‐value)
Age	60–74 years old (young old)	10.49	0.03
	75–90 years old (middle old)	31.57	
Marital status	Married	9.94	0.04
	Deceased spouse	27.58	
Type of basic insurance	Social security	11.53	0.04
	Treatment services	18.51	
	Armed forced	0	
	Banks	0	
	Petroleum	0	
Having Diabetes	Yes	16.80	0.03
	No	6.17	
The time elapsed since the initial diagnosis of the diabetes	Less than 5 years	6.25	0.04
	5–9 years	7.69	
	10–19 years	25	
	20 years and more	25	
Occurring severe complications of diabetes	Yes	32.25	0.01
	No	11.36	
Occurring visual problems due to having diabetes	Yes	50	<0.001
	No	11.65	

Abbreviation: CHE, catastrophic health expenditure.

## 4 DISCUSSION

The elderly with diabetes and hypertension incur $821 in direct medical costs annually, of which 9.7% is devoted to diagnostic services, 23.9% to visits to various therapists, and 66.4% to treatment costs. The cost of laboratory and ultrasound services contributed the most to diagnostic costs, which was expected given the noncommunicable nature of these disorders and their frequent and severe consequences. Of course, the financial burden on the elderly will diminish if these services are made available in public health clinics. The highest portion of visit costs is dedicated to general practitioners, followed by traditional healers, which can be interpreted as a lack of focus on internal specialists and endocrinologists as the primary healers of this condition. Public acceptability of traditional healers is another issue that must be addressed by educating people and organizing traditional healers. Also, medication preparation accounts for the most significant portion of treatment costs, highlighting the necessity for pharmaceutical companies to get increasingly involved in manufacturing medicines required to treat such disorders.

The study found that 12.5% of the elderly with diabetes and hypertension incur CHE. Furthermore, diabetes, hypertension, or both disorders affect 16.8%, 10.1%, and 14.5% of the elderly. Diabetes has a higher level of CHE than hypertension due to the higher cost of diagnostic and treatment services and its more severe effects (at least in the sample examined in this study). Furthermore, the intermediate level of CHE in patients with two diseases can be attributed to them receiving more supplementary insurance, having both diseases checked and controlled at the same time every time they visit the doctor and possibly better self‐care as a result of dealing with both diseases and experiencing similar risk variables.

According to research conducted by Arsenijevic (2010–2012) on the elderly with noncommunicable conditions in 15 European nations, the frequency of CHE ranges from 15% to 70%. There is a statistically significant relationship between the incidence of noncommunicable conditions like cardiovascular disease and type 2 diabetes in the elderly and the escalation of healthcare costs.^17^ According to a 2015 study by Wang et al.^1^ in China, 26% of the older population faces unaffordable medical bills. According to a 2018 study by Mobaraki et al.^2^ in Iran, CHE affect 11% of all senior people (not just those with noncommunicable conditions). The conducted study by Brinda et al.^18^ reveals that 7% of the older people in India are encountered with CHE. Also, the CHE prevalence in older people with chronic noncommunicable in Nepal is 14.6%.^19^

Examining the statistical relationship between demographic and background variables with the occurrence of CHE showed that the prevalence of CHE in young elderly individuals is low; married elderly incur fewer costs than widower elderly; and elderly covered by armed forces, banks, and oil companies incur fewer costs than elderly covered by other types of insurance. Furthermore, the elderly with diabetes incur more CHE than the elderly without diabetes; the elderly with more than 10 years of diabetes history face higher costs than the elderly with less diabetes history; and the elderly with diabetes complications, particularly visual complication, face higher costs than the elderly without these complications.

Age's impact on the severity of noncommunicable diseases and the progressive nature of these illnesses over time explain why young seniors are less likely to incur CHE. Nearly 40% of the elderly (and 80% of the research women) were homemakers who generally had no independent income. This may explain why the prevalence of CHE is lower in married seniors. Health insurance provided by the armed forces, banks, and oil corporations reduced CHE for the elderly covered by these plans. This improvement was linked to the three elements of universal health coverage (population coverage, services, and costs). It is no surprise that the high cost of diabetes and its consequences is linked to the increased likelihood of CHE in diabetic people. Due to the noncommunicable and age‐related nature of diabetes, people with a history of the condition longer than 10 years are more likely to incur CHE. CHE are more common among diabetic patients suffering from disease complications, such as visual impairment. This is because these complications are more difficult to cure and have a more detrimental effect on the elderly's capacity to work and earn a living, increasing healthcare costs.

Wang et al.'s^1^ research in China demonstrates how two or more noncommunicable diseases (co‐morbidity) are associated with a dramatic increase in CHE. Mobaraki et al.^2^ found that factors like household income, household size, senior employment, and supplemental health insurance all had a significant impact on the likelihood of experiencing CHE.

In light of the study's findings, the authors propose the following measures to lower the costs associated with providing medical care to the elderly: improving access to healthcare for the elderly by expanding coverage under existing health plans and introducing new ones; public health clinics' provision of laboratory and sonographic services; the organization and definition of the medical scope of traditional healers; the dissemination of information regarding the significance of controlling diabetes and its complications and the negative consequences of neglecting professional medical care; increasing access to internists and endocrinologists for the elderly through the HMO; Supporting knowledge enterprises in supplying medicines needed by the elderly suffering from such diseases; focusing on preventing the development of noncommunicable diseases in old age; better management of the disease by continuous follow‐up to prevent the development of complications; and providing practical in‐service training for general practitioners to treat diabetes and hypertension more effectively. Comparable studies should be undertaken in other regions to extract the national pattern of elderly costs. Similar studies should be conducted in Semnan regularly to follow future trends and evaluate the efficiency of promotional actions.

The study's dependence on the elderly's memory in recalling care costs was a significant flaw that possibly created recall bias. To overcome this problem, an effort was made to ask knowledgeable family members about the costs imposed on the elderly. The lack of a comprehensive health information system to track the type and amount of health care received, as well as the costs incurred by the elderly, is the source of this constraint.

## 5 CONCLUSION

Compared to other sectors and studies, the rate of CHE among the elderly with diabetes and hypertension is quite acceptable. However, there is still room for improvement with appropriate promotional strategies. Effectively enhancing the efficacy of these treatments can be accomplished by focusing on preventative measures and paying specific attention to the elderly who have experienced increased exposure. The purpose of a research study is to inform decision‐making organizations about an issue by describing the current situation and proposing feasible solutions so that the findings can serve as a guide for health officials.

## AUTHOR CONTRIBUTIONS

**Sayed Amir Masoud Meshkat**: Conceptualization; data curation; investigation; methodology; validation; writing—original draft. **Sayed Saeed Kassaeian**: Conceptualization; formal analysis; investigation; methodology; software; validation; writing—review & editing. **Navid Danaei**: Conceptualization; data curation; formal analysis; investigation; methodology; software; validation; writing—review & editing. **Farid Gharibi**: Conceptualization; data curation; formal analysis; investigation; methodology; project administration; software; supervision; validation; visualization; writing—original draft.

## CONFLICT OF INTEREST STATEMENT

The authors declare no conflict of interest.

## TRANSPARENCY STATEMENT

The lead author Farid Gharibi affirms that this manuscript is an honest, accurate, and transparent account of the study being reported; that no important aspects of the study have been omitted; and that any discrepancies from the study as planned (and, if relevant, registered) have been explained.

## Data Availability

Data are stored in the university of study origin. If any researcher is interested, for valid reason, they may contact the corresponding author (F. G.), gharibihsa@gmail.com.
